# Strawberry and cranberry polyphenols improve insulin sensitivity in insulin-resistant, non-diabetic adults: a parallel, double-blind, controlled and randomised clinical trial

**DOI:** 10.1017/S0007114517000393

**Published:** 2017-03-14

**Authors:** Martine Paquette, Ana S. Medina Larqué, S. J. Weisnagel, Yves Desjardins, Julie Marois, Geneviève Pilon, Stéphanie Dudonné, André Marette, Hélène Jacques

**Affiliations:** 1 Institute of Nutrition and Functional Foods, Laval University, Quebec, Canada, G1V 0A6; 2 School of Nutrition, Laval University, Quebec, Canada, G1V 0A6; 3 Diabetes Research Unit, Endocrinology and Nephrology Axis, Research Centre, Laval University Health Center of Quebec, Quebec, Canada, G1V 4G2; 4 Quebec Heart and Lung Institute, Quebec, Canada, G1V 4G5

**Keywords:** Polyphenols, Strawberries, Cranberries, Insulin sensitivity, Glucose metabolism, Insulin secretion, Insulin-resistant subjects

## Abstract

Plant-derived foods rich in polyphenols are associated with several cardiometabolic health benefits, such as reduced postprandial hyperglycaemia. However, their impact on whole-body insulin sensitivity using the hyperinsulinaemic-euglycaemic clamp technique remains under-studied. We aimed to determine the effects of strawberry and cranberry polyphenols (SCP) on insulin sensitivity, glucose tolerance, insulin secretion, lipid profile, inflammation and oxidative stress markers in free-living insulin-resistant overweight or obese human subjects (*n* 41) in a parallel, double-blind, controlled and randomised clinical trial. The experimental group consumed an SCP beverage (333 mg SCP) daily for 6 weeks, whereas the Control group received a flavour-matched Control beverage that contained 0 mg SCP. At the beginning and at the end of the experimental period, insulin sensitivity was assessed by a hyperinsulinaemic-euglycaemic clamp, and glucose tolerance and insulin secretion by a 2-h oral glucose tolerance test (OGTT). Insulin sensitivity increased in the SCP group as compared with the Control group (+0·9 (sem 0·5)×10^−3^ *v*. −0·5 (sem 0·5)×10^−3^ mg/kg per min per pmol, respectively, *P*=0·03). Compared with the Control group, the SCP group had a lower first-phase insulin secretion response as measured by C-peptide levels during the first 30 min of the OGTT (*P*=0·002). No differences were detected between the two groups for lipids and markers of inflammation and oxidative stress. A 6-week dietary intervention with 333 mg of polyphenols from strawberries and cranberries improved insulin sensitivity in overweight and obese non-diabetic, insulin-resistant human subjects but was not effective in improving other cardiometabolic risk factors.

According to the International Diabetes Federation^(^ ^1^ ^)^, up to 592 million people worldwide (one in ten adults) will suffer from type 2 diabetes by the year 2035. This alarming increase has been predicted based on several factors, such as the high prevalence of obesity and sedentary lifestyles^(^ ^2^ ^,^ ^3^ ^)^. Indeed in obese humans, elevated levels of NEFA, pro-inflammatory cytokines and other factors produced by adipose tissue are key determinants of insulin resistance^(^ ^4^ ^)^. To maintain plasma glucose at normal levels, pancreatic *β*-cells must adjust their function to compensate for insulin resistance. This leads to an exhaustion of *β*-cell insulin secretion and the development of impaired glucose tolerance (IGT) and subsequent type 2 diabetes.

In recent decades, scientific evidence has shown a link between increased consumption of fruits and vegetables, particularly berries, and reduced incidence of type 2 diabetes^(^ ^5^ ^)^. Recent reviews have indeed reported that berries, like strawberries and cranberries, can lower markers of cardiometabolic risk^(^ ^6^ ^–^ ^11^ ^)^ and improve markers of the metabolic syndrome in humans^(^ ^12^ ^–^ ^14^ ^)^. It is well-documented that strawberries and cranberries are rich in polyphenols and contain a wide variety of phenolic compounds, ranging from phenolic acids (hydroxybenzoic and hydroxycinnamic acids), flavonoids (anthocyanins, flavonols and flavan-3-ols) to polymerised molecules (proanthocyanidins and ellagitannins)^(^ ^15^ ^)^. According to several *in vitro* and animal studies, polyphenols may improve glucose metabolism^(^ ^16^ ^)^ and peripheral glucose uptake in insulin-sensitive tissues by increasing GLUT4 translocation and activity and reducing oxidative stress and inflammation^(^ ^17^ ^,^ ^18^ ^)^. It has recently been demonstrated that anthocyanin-rich bilberry extract reduces glycaemia and improves insulin sensitivity in diabetic mice^(^ ^19^ ^)^. Afrin *et al.* ^(^ ^12^ ^)^ reviewed six human intervention studies on the impact of strawberries on the metabolic syndrome, and specifically on the prevention of type 2 diabetes, but none used the gold standard clamp technique to assess insulin sensitivity.

There are several indices calculated from the oral glucose tolerance test (OGTT), such as the Matsuda index and insulin sensitivity index (ISI), or from fasting glycaemia and/or insulinaemia, such as the homeostasis model assessment of insulin sensitivity (HOMA-IR) and the quantitative insulin sensitivity check index (QUICKI), that indirectly estimate insulin sensitivity in humans. However, these indices are not as accurate as a direct measurement of whole-body insulin sensitivity by the hyperinsulinaemic-euglycaemic clamp^(^ ^20^ ^)^. In this respect, the clamp technique is recognised as the reference method for measuring insulin sensitivity, and according to Antuna-Puente *et al.* ^(^ ^21^ ^)^, it should be promoted in clinical human studies. So far, only two studies have used a hyperinsulinaemic-euglycaemic clamp to assess the effect of berry polyphenols on insulin sensitivity^(^ ^22^ ^,^ ^23^ ^)^. According to one study in which obese non-diabetic insulin-resistant participants received a blueberry or placebo smoothie twice a day, the mean percentage increase in insulin sensitivity was five times greater in the experimental group compared with that in the placebo group^(^ ^22^ ^)^. In the second study, a grape polyphenol supplement protected against a decrease in insulin sensitivity caused by a fructose-rich diet in overweight subjects^(^ ^23^ ^)^. To the best of our knowledge, there are no human studies evaluating the effects of strawberry and cranberry polyphenols (SCP) on insulin sensitivity assessed by the hyperinsulinaemic-euglycaemic clamp in non-diabetic insulin-resistant subjects.

The proposed study aims to test the effects of an SCP blend incorporated in a beverage on insulin sensitivity and related parameters in free-living overweight or obese men and women with insulin resistance. This blend has been comprehensively analysed and contains well-defined amounts of anthocyanins, proanthocyanidins, ellagitannins, phenolic acids and flavonols (quercetins)^(^ ^24^ ^)^. The primary endpoint was the difference in the change in insulin sensitivity after 6 weeks of SCP consumption compared with a SCP-free Control beverage. Secondary endpoints aimed to assess changes in glucose tolerance, insulin secretion, plasma lipids, markers of oxidative stress and inflammation, as well as to characterise plasma phenolic components as bioavailability (efficacy) outcomes. We hypothesised that the consumption of SCP improves insulin sensitivity and lipid profile and reduces inflammatory and oxidative stress markers in overweight/obese subjects.

## Methods

### Study design

This 6-week clinical trial was a randomised, double-blinded, controlled, parallel study performed at the Institute of Nutrition and Functional Foods (INAF) in Quebec City , Canada. The study was conducted in accordance with the Declaration of Helsinki and all procedures involving human subjects were approved by the Research Ethical Committee of the Quebec University Health Center. Written informed consent was obtained from all participants after reading a detailed consent form prior to their participation in the study. This trial was registered at clinicaltrials.gov as NCT01766570. The study was conducted, including analyses, between spring 2012 and fall 2015.

### Subjects

All subjects were overweight or obese (BMI≥25 kg/m^2^) and insulin resistant based on fasting plasma insulin level >60 pmol/l (J.-P. Després and J. Bergeron, unpublished results). Subjects may have also displayed impaired fasting plasma glucose (IFG) (5·6–6·9 mmol/l) with or without IGT (7·8–11·0 mmol/l) following a 2-h 75 g OGTT^(^ ^25^ ^)^. Exclusion criteria included smoking, chronic disease (diabetes, respiratory, renal, gastrointestinal or hepatic disease, CVD, hypertension, cancer), metabolic or acute disease, use of medication or supplement known to affect lipid or glucose metabolism, use of antioxidant supplements, major surgery in the 3 months preceding the study and significant weight change (SEM 10 %) within 6 months prior to beginning the study.

A total of 116 subjects, recruited in the Quebec City metropolitan area by media advertising, were first screened to examine their eligibility to participate in this study. Of the fifty eligible subjects who began the experimental period, forty-six participants (twenty men and twenty-six women) aged between 40 and 70 years completed the study. However, five subjects (three in the SCP group and two in the Control group) were excluded from analysis because they no longer met some inclusion criteria, leading to a total of eighteen men (nine in each of the two groups) and twenty-three postmenopausal women (eleven in the SCP group and twelve in the Control group) who were included in the statistical analysis (online Supplementary Fig. S1).

### Experimental groups

The polyphenol blend and Control were provided as energy-free beverages formulated with purified water and small amounts of sucralose. Red food colour was added in the Control beverage. Both beverages thus had the same taste and visual aspect. Both were formulated in a single batch by Atrium Innovations Inc. and were provided in dark bottles at 120 ml/d. The bottles were sealed and kept at room temperature with limited light exposure. The polyphenol blend contained 1·84 g of a mixture of dry strawberry (*Fragaria×ananassa Duch*) and cranberry (*Vaccinium macrocarpon* L.) polyphenol extracts (GlucoPhenol^TM^; NutraCanada) providing an average daily dose of 333 (SD 12) mg of polyphenols (18 % total polyphenols in GlucoPhenol^TM^, as determined by Folin–Ciocalteu assay). SCP (GlucoPhenol^TM^) complies with good manufacturing practices according to Health Canada regulations. To determine the amount of polyphenols to be used for the present study, we first conducted a bioavailability study in rats with different doses of SCP^(^ ^24^ ^)^. The dose of 333 mg polyphenols was chosen as corresponding, for a 60 kg human, to the dose (36 mg/kg) that induced the highest concentration of plasma total phenolic metabolites in the rat^(^ ^24^ ^)^. The total phenolic content was monitored in the SCP beverage throughout the study, ensuring a minimum of 300 mg/d. In order to simulate the taste and colour of the SCP-containing beverage, a pomegranate-derived red food colour was used in the SCP-free Control beverage providing a small quantity of polyphenols. The two beverages were characterised for their phenolic composition as previously described^(^ ^24^ ^)^ (Table 1). In brief, proanthocyanidins were analysed by normal-phase HPLC with fluorescence detection, and quantified using an epicatechin standard. Phenolic acids were analysed using reverse-phase ultra-high performance liquid chromatography (UHPLC) coupled with tandem MS and their quantification was achieved using their corresponding standard when available, or their aglycone or most similar phenolic structure otherwise. The daily phenolic dose provided by SCP corresponds approximately to the intake of 112 g of fresh fruit (equivalent to two servings).

**Table 1:** Phenolic composition of experimental beverages (Mean values and standard deviations)

	SCP		Control	
Phenolic composition (µg/120 ml)	Mean	sd	Mean	sd
Proanthocyanidins	20 040*	522	5070	169
Monomers	4156*	9	1276	36
Dimers	237	5	ND	ND
Polymers	15 647*	528	3794	133
Phenolic acids	28 206*	256	5013	138
Gallic acid	728*	5	11	2
Hydroxybenzoic acid	3334*	94	103	12
*p*-Coumaric acid	9095*	171	47	9
*m*-Coumaric acid	2051*	80	6	1
Caffeic acid	372	22	ND	ND
Ferulic acid	65*	7	33	4
Vanillic acid	107*	3	3	0·3
Caffeoyl glucoside	1282*	54	8	2
Coumaroyl glucoside	5508*	178	9	4
Chlorogenic acid	927*	14	240	28

SCP, strawberry and cranberry polyphenols; ND, not detectable/below limit of detection. The SCP beverage provided an average daily dose of 333 (sd 12) mg of polyphenols, as determined by Folin–Ciocalteu assay.

* Welch’s *t* test and the Mann–Whitney test showed significant differences (*P*<0·05) in concentrations of each measured phenolic compound in SCP beverage compared with Control.

### Study timeline

Participants were equally divided into two groups by sex after a 2-week run-in period. During this period, subjects were asked to maintain their usual food habits and physical activity level, and were limited to one unit drink or less of beer or spirits per day. The consumption of berries, wine, polyphenol supplements and all products containing berries or wine was also forbidden. Assignment of treatment was conducted through the use of a random sequence of numbers. Allocation to treatment was concealed by a secure computer-assisted method enabling preservation of assignments until enrolment was assured and confirmed. Men and women were equally distributed among the two groups using the same computer-assisted method. The study sponsor held the trial codes which were disclosed after completion of the statistical analyses. Participants in the treatment group consumed an SCP-containing beverage, whereas the Control group received a flavour-matched SCP-free Control beverage, daily for a 6-week period. During the experimental period, subjects were asked to follow the same aforementioned recommendations as during the run-in period. Approximately 2–3 weeks after the beginning of the experimental period, a registered dietitian called all participants once to ensure that they consumed the beverages daily and followed the prescribed instructions. Subjects were instructed to shake the bottle before consumption and drink the beverage with or without food regardless of the time of day. To document compliance, subjects were requested to bring back the unused bottles at the end of the study. Bottle counts indicated a 99 % compliance in both groups. Also, a 6-week checklist was provided to all participants to identify study materials that had not been ingested, thus providing us with a tool to confirm the compliance rate of the participants.

### Anthropometric and blood pressure measurements

Fasted body weight, height, waist and hip circumferences were measured at the beginning and at the end of the study using standardised methods. BMI and waist:hip ratio were then calculated. Blood pressure was measured three times on the right arm with an automatic tensiometer (Digital Blood Pressure Monitor, model HEM-907XL; OMRON^®^) following a 10-min rest at the beginning and at the end of the experimental period.

### Food records and questionnaires

During the screening visit, two online self-administered questionnaires were completed by all subjects to collect information on medical history, lifestyle, economic and socio-demographic characteristics. Participants were also asked to complete two online self-administered questionnaires at the beginning and at the end of the experimental period, including a validated FFQ to record energy and macronutrient intake for 28 consecutive days^(^ ^26^ ^)^ and a short physical activity questionnaire. There was also an additional questionnaire on the subjects’ liking of the experimental beverages (taste, texture, etc.) and on side effects, administered at the end of the study. Changes in medication, temporary medication, natural health products intake or consumption of any other food supplements were monitored according to the exclusion criteria during the entire study period.

### Hyperinsulinaemic-euglycaemic clamp

A 120-min hyperinsulinaemic-euglycaemic clamp was performed at the beginning and at the end of the experimental period at the Diabetes Research Unit of the Laval University Health Center of Quebec after a 12-h overnight fast, according to the method described by Piche *et al*.^(^ ^27^ ^)^. Alcohol intake was forbidden 48 h before the clamp. Insulin sensitivity (*M/I*) was calculated from glucose infusion rate (mg/min) during the final 30 min of the clamp divided by body weight (kg) and then divided by the mean insulin concentration during the final 30 min of the clamp (mg/kg per min per pmol)^(^ ^27^ ^)^. For NEFA analysis, additional blood samples were collected during the clamp at 0, 30, 60, 90 and 120 min, centrifuged after 30 min at room temperature and then stored at −80°C until analysis.

### Oral glucose tolerance test

A 75-g OGTT was performed 2–3 d before each clamp at the beginning and at the end of the experimental period at INAF to assess glucose tolerance after a 12-h overnight fast. Blood samples were collected at time points −15, 0, 15, 30, 60 and 120 min, immediately centrifuged and kept at −20°C for further measurements of glucose, insulin and C-peptide. Alcohol intake was forbidden 48 h before the test. For the second clamp and OGTT, participants were asked to consume the beverage 12 h before their appointment.

### Blood collection and storage

Plasma and serum samples were collected in the fasting state, before each OGTT and clamp, and stored at −80°C for further analysis of lipids, inflammatory markers (high-sensitivity C-reactive protein (hsCRP), IL-6, TNF-*α*, high molecular weight (HMW) adiponectin and regulated on activation normal T cell expressed and secreted (RANTES)/chemokine ligand 5 (CCL5)), plasminogen activator inhibitor-1 (PAI-1), a marker of cardiovascular risk, and ferric reducing antioxidant power (FRAP) and oxidised LDL, markers of oxidative stress.

### Glucose, insulin and C-peptide

Plasma glucose was determined using an enzymatic method^(^ ^28^ ^)^ and plasma insulin was measured by RIA with polyethylene glycol separation^(^ ^29^ ^)^. Plasma C-peptide level, an indicator of insulin secretion used to estimate pancreatic *β*-cell function, was determined using a modified version of the method of Hedging with polyclonal antibody A-4741 from Ventrex and polyethylene glycol precipitation^(^ ^29^ ^)^.

### Lipids

LDL and HDL were isolated from fresh blood by ultracentrifugation combined with a heparin–manganese chloride precipitation^(^ ^30^ ^)^. Then, cholesterol and TAG concentrations in total serum and lipoproteins were determined enzymatically by using a Technicon RA-500 analyzer (Bayer). NEFA were determined in serum via an enzymatic colorimetric assay (Wko Diagnostics) using a Beckman Olympus AU400 (Beckman Coulter Canada LP).

### Inflammatory, thrombogenic and oxidative markers

Serum level of hsCRP was measured using nephelometry as described previously^(^ ^31^ ^)^. PAI-1, IL-6 and TNF-*α* were measured in plasma at the Quebec Heart and Lung Institute, Quebec, using commercially available Multiplex kits (EMD Millipore). Plates were read and analysed using the Bio-Plex 200 system (Bio-Rad). Oxidised-LDL, HMW adiponectin and RANTES were determined using a commercially available ELISA (Mercodia; R&D Systems) according to manufacturer’s instructions. Total antioxidant capacity of plasma, assessed by FRAP assay, was determined as described previously^(^ ^32^ ^)^.

### Bioavailability study

A subgroup of seventeen subjects performed an additional bioavailability study, to identify circulating phenolic metabolites following the administration of experimental beverages. Halfway through the supplementation period (30 (sem 3) d), fasted subjects were administered their respective treatment at INAF (SCP, *n* 8; Control, *n* 9). Blood samples were collected using EDTA-containing syringes before and 30, 60, 120, 240 and 360 min after the ingestion. During the experiment, all subjects were kept fasted. Plasma samples were obtained by centrifugation (3500 rpm, 10 min at 4°C). Plasma phenolic compounds were characterised by UHPLC–MS/MS as previously described^(^ ^24^ ^)^, with slight modifications. Acidified plasma samples (300 µl) were loaded into preconditioned Waters OASIS HLB (Waters Ltd) µElution plates 2 mg–30 µm. The retained phenolic compounds were eluted with 75 µl of acetone–ultrapure water–acetic acid solution (70:29·5:0·5, v/v/v) in presence of rosmarinic acid as internal standard (1 µg/ml final concentration). The eluted solutions were directly analysed by UHPLC–MS/MS, using a Waters Xevo TQD MS (Waters Ltd) coupled to a Waters Acquity UHPLC (Waters Ltd). Phenolic metabolites were separated and identified as previously reported^(^ ^24^ ^)^.

### Statistical analyses

We estimated sample size based on the primary endpoint of insulin sensitivity from data published by Stull *et al.* ^(^ ^22^ ^)^ and Ouellet *et al.* ^(^ ^33^ ^)^. For this purpose, we used the following values: an average difference in changes from baseline (Post *v*. Pre) of 17×10^−3^ mg/kg per min per pmol for insulin sensitivity between the SCP and Control groups after 6 weeks and an estimated sd of 18. Power calculation at 80 % with a two-sided significance level set at 0·05 showed that a minimum of forty subjects, twenty in each group, was required to observe significant changes from baseline in insulin sensitivity between the SCP and Control groups over a 6-week dietary intervention, taking into account 25 % expected dropouts. Statistical analyses were performed using SAS 9.3 (SAS Institute).

Paired *t* tests were performed to compare changes from baseline (Post *v*. Pre) within the same group. Because baseline insulin sensitivity was correlated with *M/I* (*r*_s_=0·50; *n* 39; *P*<0·001) only, PROC MIXED for ANCOVA with baseline insulin sensitivity as covariate was used to compare the changes in *M/I* between the two treatments. A two-way repeated-measures ANOVA was applied for variables with repeated measures over time (glucose, insulin, C-peptide and NEFA concentrations during the OGTT or clamp). In this model, no significant time-by-treatment interaction was observed. Furthermore, positive incremental AUC (IAUC) for glucose (mmol/l per min), insulin (pmol/l per min) and C-peptide (pmol/l per min) up to 30 and 120 min were calculated using the trapezoid method with baseline value corresponding to the fasting level (time point −15 min of the OGTT). The percentage change in IAUC ((IAUC post value−IAUC pre value)×100/IAUC pre value) was also calculated. PROC MIXED for a two-way ANOVA was used to compare changes from baseline for anthropometric and blood pressure measurements, IAUC glucose, insulin and C-peptide during OGTT, lipid and cardiovascular parameters and markers of inflammation and oxidative stress. Data from men and women were pooled together because there was no significant sex-by-treatment interaction. As several significant correlations were observed within and between OGTT and lipid variables, a Bonferroni correction was applied, setting the two-sided significance level at *P*<0·004 for those variables. No Bonferroni correction was applied for *M/I,* inflammatory and oxidative stress because no correlation was observed between those and the other variables, and a two-sided significance level was set at 0·05. PROC GLM ANOVA was used to compare the FFQ variables.

Plasma concentrations of phenolic metabolites were measured post-ingestion in a subgroup (*n* 17) and were compared using the Welch’s *t* test (correcting for unequal variance) when data were assumed to be normally distributed (or met the criteria for normality after log transformation), or using the non-parametric Mann–Whitney test otherwise, using GraphPad Prism 6.05 software. Correlations were assessed between circulating concentrations of phenolic metabolites (expressed as area under the plasma concentration (nm) time (min) curve between 0 and 30 min after the ingestion of the beverages) and changes in *M/I* and in outcomes of OGTT (expressed as the percentage change in the IAUC for glucose, insulin and C-peptide during the first 30 min of the OGTT). A robust regression with *M*-estimations was adjusted using the procedure ROBUSTREG of SAS 9.4. To perform an optimal selection of the model, the smallest Akaike criterion was deemed the best model. Considering the small size of the subgroup and the multiplicity of analysis, a Bonferroni correction (*P*<0·0025) was performed for post-ingestion plasma concentration of phenolic metabolites. Bonferroni correction was also applied for the correlational analysis with the outcomes of the first 30 min OGTT parameters (*P*<0·017). Since *M/I* assesses insulin sensitivity by hyperinsulinaemic-euglycaemic clamp, correlations with *M/I* changes were analysed separately from the OGTT rate changes and remained at a statistically significant level of *P*≤0·05. The results are presented as means with their standard errors.

## Results

### Subject baseline characteristics

Baseline clinical and laboratory characteristics of participants are shown in Table 2. All subjects were insulin resistant, overweight or obese (BMI≥25 kg/m^2^) with increased abdominal adiposity (waist circumference >94 cm for men and >80 cm for women). There were no differences between the two groups regarding age, body weight, BMI, waist and hip circumferences, lipid profile, fasting plasma glucose, 2-h plasma glucose or fasting plasma insulin.

**Table 2:** Baseline characteristics of the study participants* (Mean values with their standard errors; numbers and percentages)

	SCP (*n* 20)		Control (*n* 21)		
Variables	Mean	sem	Mean	sem	*P*
Age (years)	57	1	60	1	0·18
Body weight (kg)	85	3	85	3	0·97
BMI (kg/m^2^)	31	1	31	1	0·91
Waist circumference (cm)	104	3	104	2	0·95
Hip circumference (cm)	111	2	111	2	0·93
Cholesterol (mmol/l)					
Total	5·70	0·17	5·37	0·22	0·07
HDL	1·25	0·05	1·33	0·05	0·24
LDL	3·52	0·17	3·20	0·15	0·18
Total TAG (mmol/l)	2·03	0·24	1·73	0·26	0·39
Total cholesterol:HDL-cholesterol ratio	4·8	0·3	4·1	0·2	0·07
Fasting plasma glucose (mmol/l)	6·0	0·1	5·8	0·1	0·16
2-h plasma glucose (mmol/l)	7·7	0·4	7·4	0·4	0·71
Fasting plasma insulin (pmol/l)	118	11	130	11	0·45
	*n*	%	*n*	%	
Sex					
Women	11	55	12	57	–
Men	9	45	9	43	–

SCP, strawberry and cranberry polyphenols.

* PROC MIXED ANOVA test showed no significant differences in baseline characteristics between the two groups.

At baseline, all subjects had a high fasting plasma insulin level (>60 pmol/l), of whom thirty-one had fasting plasma insulin levels >90 pmol/l. From data collected during the pre-intervention OGTT and according to the Expert Committee on the Diagnosis and Classification of Diabetes Mellitus^(^ ^25^ ^)^, twelve subjects had both IFG (5·6–6·9 mmol/l) and IGT (7·8–11·0 mmol/l), seventeen subjects had IFG only, three subjects had IGT only and nine among them had normal glucose tolerance (fasting plasma glucose <5·6 mmol/l and plasma glucose <7·8 mmol/l after 120 min).

### Food intake and physical activity

According to FFQ data (online Supplementary Table S1), there were no differences in baseline dietary intake or in changes from baseline (Post *v*. Pre) in energy and macronutrient intake between the two groups. In addition, no change from the level of physical activity was perceived during the study (data not shown). As for side effects, no major harmful or unexpected effects were reported in either group.

### Anthropometric measurements and blood pressure

Body weight, anthropometric, systolic and diastolic blood pressure measurements were performed at the beginning and at the end of the experimental period. No differences in changes from baseline (Post *v*. Pre) were observed for these parameters between the two groups (online Supplementary Table S2).

### Insulin sensitivity and other parameters of glucose homoeostasis

**Fig. 1:**
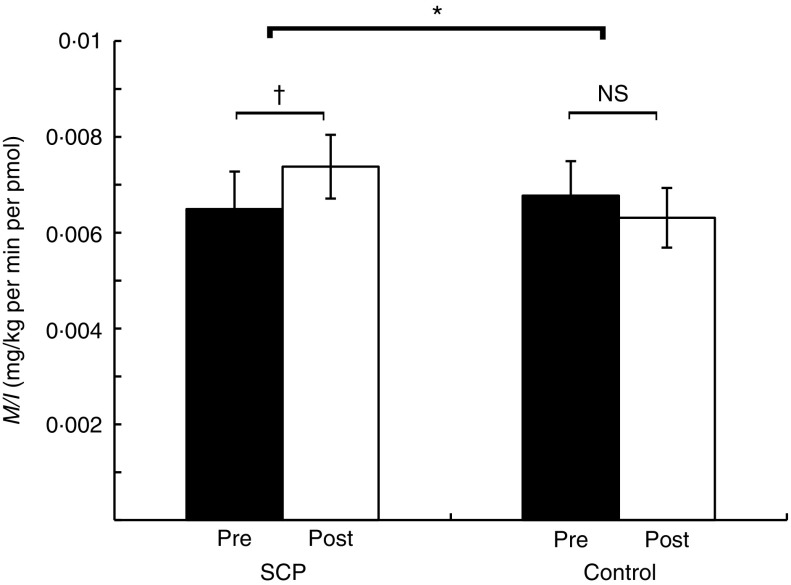
Insulin sensitivity (*M/I*) before (Pre) and after (Post) 6-week consumption of strawberry and cranberry polyphenols (SCP) or Control in insulin-resistant human subjects. Values are means (*n* 39), with their standard errors represented by vertical bars. NS, no significant difference. Paired *t* test for comparisons from baseline (Post *v*. Pre) within each group showed a significant increase with SCP, † *P*=0·05. PROC MIXED for ANCOVA with baseline insulin sensitivity as covariate indicated significant difference in changes from baseline (Post *v*. Pre) between the two groups, * *P*=0·03.

Insulin sensitivity (*M/I*) increased by 14 % (+0·9 (sem 0·5)×10^−3^ mg/kg per min per pmol) (Fig. 1) in the SCP group (*P*=0·05), whereas it decreased by 7 % (−0·5 (sem 0·5)×10^−3^ mg/kg per min per pmol) in the Control group without achieving a level of significance (*P*=0·28). When we compared the changes from baseline (Post *v*. Pre) between the two treatments, the SCP group showed significant improvement in insulin sensitivity (*P*=0·03) compared with Control.

**Fig. 2:**
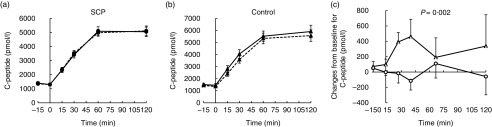
Responses of plasma C-peptide at −15, 0, 15, 30, 60 and 120 min during the oral glucose tolerance test (OGTT) before (Pre) and after (Post) 6-week consumption of (a) strawberry and cranberry polyphenols (SCP) or (b) Control, in insulin-resistant human subjects. (c) Changes from baseline (Post *v*. Pre) in plasma C-peptide at −15, 0, 15, 30, 60 and 120 min during the OGTT before (Pre) and after (Post) 6-week consumption of SCP or Control in insulin-resistant human subjects. Repeated measures ANOVA showed significant difference in the changes from baseline (Post *v*. Pre) between the two groups over time during the OGTT in insulin-resistant human subjects. A Bonferroni correction was applied defining level of statistical significance at *P*<0·004. Values are means (*n* 41), with their standard errors represented by vertical bars. 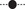, SCP Pre values; 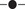, SCP Post values; 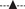, Control Pre values; 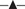, Control Post values; 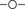, SCP (Post *v*. Pre values); 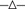, Control (Post *v*. Pre values).

We also performed repeated measurements ANOVA for glucose (Table 3), insulin (Table 3) and C-peptide (Fig. 2(a) and (b)) up to 120 min during OGTT and for NEFA (online Supplementary Fig. S2) over time during the clamp. There were no differences between baseline values (Pre) for all glucose metabolism parameters. Whereas glucose, insulin and NEFA responses were not different between treatments, there was an overall increase in C-peptide with Control compared with SCP (*P*=0·002) (Fig. 2(c)).

**Table 3:** Incremental AUC (IAUC) and time point values over time during oral glucose tolerance test (OGTT) for glucose and insulin before and after 6-week consumption of strawberry and cranberry polyphenols (SCP) or Control in insulin-resistant human subjects (Mean values with their standard errors)

	SCP (*n* 20)					Control (*n* 21)					
	Pre		Post			Pre		Post			
Variables	Mean	sem	Mean	sem	*P*_1_ *	Mean	sem	Mean	sem	*P*_2_ *	*P* †
IAUC glucose up to 30 min (mmol/l per min)	56	4	58	6	0·77	55	5	53	4	0·48	0·77
Plasma glucose (mmol/l)					0·42					0·53	0·31‡
−15	6·1	0·1	6·1	0·1		5·9	0·1	6·0	0·1		
0	6·0	0·1	6·1	0·1		5·8	0·1	5·9	0·1		
15	8·1	0·2	8·1	0·3		7·7	0·2	8·0	0·2		
30	9·7	0·3	9·8	0·3		9·3	0·3	9·4	0·3		
60	10·3	0·5	10·6	0·4		9·9	0·5	9·5	0·4		
120	7·7	0·4	7·5	0·4		7·4	0·4	6·9	0·3		
IAUC glucose up to 120 min (mmol/l per min)	348	31	357	32	0·71	329	34	291	27	0·11	0·16
IAUC insulin up to 30 min (pmol/l per min)	9	1	8	1	0·56	10	1	12	2	0·12	0·13
Plasma insulin (pmol/l)					0·41					0·34	0·21‡
−15	129	11	132	14		134	12	144	16		
0	118	9	120	13		130	13	131	17		
15	402	37	418	53		479	58	600	85		
30	729	73	645	76		759	79	822	106		
60	1006	99	968	104		1173	148	1064	127		
120	895	111	818	99		1094	184	1208	233		
IAUC insulin up to 120 min (pmol/l per min)	80	8	74	8	0·40	95	12	95	13	0·94	0·51

* *P* value obtained from paired *t* test to compare changes from baseline (Post *v*. Pre) within the SCP (*P*_1_) and Control (*P*_2_) groups.

† *P* value represents between-treatment comparison of changes from baseline (Post *v*. Pre), assessed by PROC MIXED ANOVA.

‡ *P* value obtained from repeated measures ANOVA performed on the averages of all time points for SCP and Control to assess treatment effect. Due to significant correlations within OGTT variables, a Bonferroni correction was applied for these variables, defining level of statistical significance at *P*<0·004.

**Fig. 3:**
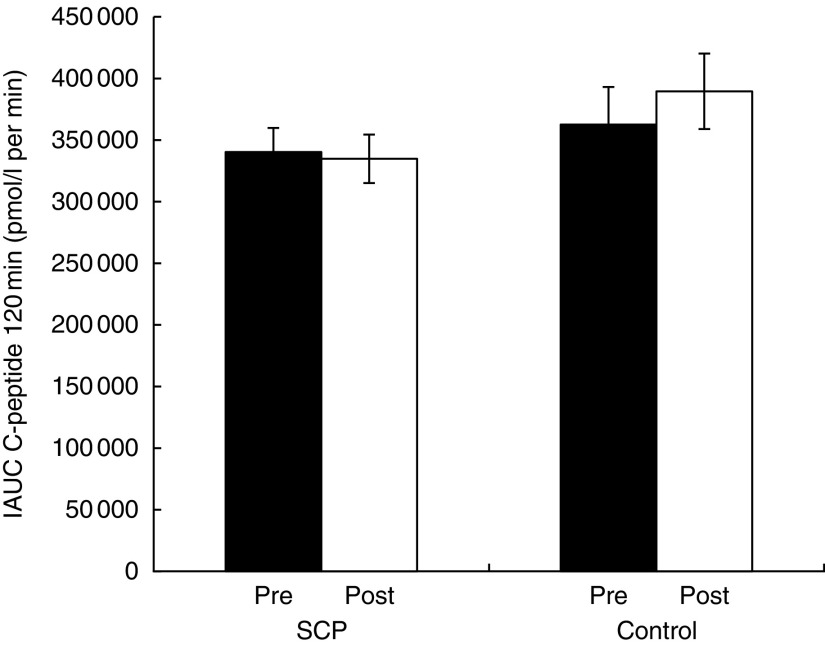
Positive incremental AUC (IAUC) up to 120 min of the oral glucose tolerance test for C-peptide concentrations before (Pre) and after (Post) 6-week consumption of strawberry and cranberry polyphenols (SCP) or Control in insulin-resistant human subjects. Values are means (*n* 41), with their standard errors represented by vertical bars. Paired *t* test showed no difference in changes from baseline (Post *v*. Pre) in the SCP and Control groups. PROC MIXED for a two-way ANOVA showed no significant effect in the changes from baseline (Post *v*. Pre) between the two groups.

**Fig. 4:**
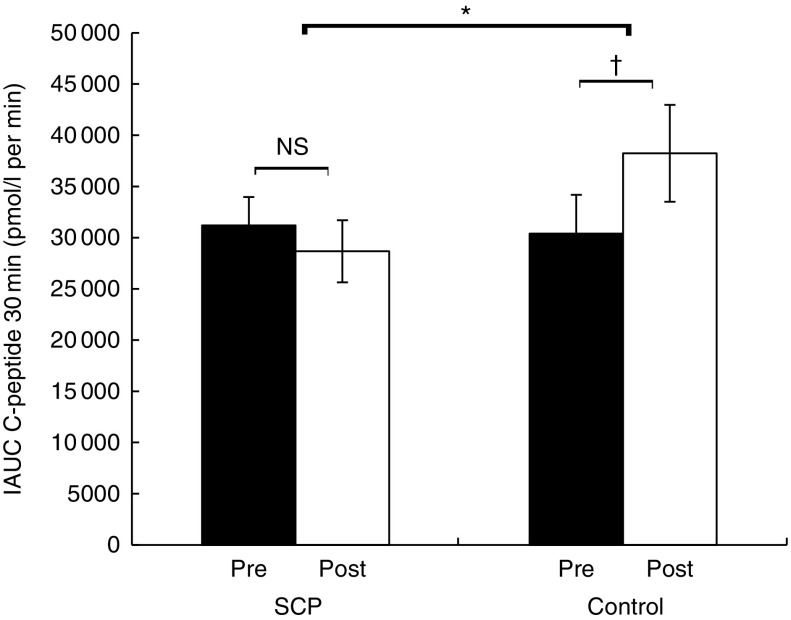
Positive incremental AUC (IAUC) up to 30 min of the oral glucose tolerance test for C-peptide concentrations before (Pre) and after (Post) 6-week consumption of strawberry and cranberry polyphenols (SCP) or Control in insulin-resistant human subjects. Values are means (*n* 41), with their standard errors represented by vertical bars. NS, no significant difference. Paired *t* test for comparisons from baseline (Post *v*. Pre) within each group showed a significant increase with Control, † *P*=0·003. PROC MIXED for a two-way ANOVA showed a significant difference in the changes from baseline (Post *v*. Pre) between the two groups, * *P*=0·002.

The mean IAUC up to 30 min corresponding to the early phase of insulin response during the OGTT and the mean IAUC up to 120 min after the OGTT are shown in Table 3 for plasma glucose and insulin and in Fig. 3 and 4 for C-peptide. No differences in changes from baseline (Post *v*. Pre) for plasma IAUC glucose (Table 3), IAUC insulin (Table 3) and IAUC C-peptide (Fig. 3) were observed up to 120 min within each group or between the two groups. However, compared with the baseline (Pre) values, plasma IAUC C-peptide up to 30 min was increased by 26 % in the Control group (*P*=0·003) and non-significantly reduced by 8 % in the SCP group (*P*=0·21). These changes were different between the two groups (*P*=0·002) (Fig. 4).

### Lipid profile

No differences in changes from baseline (Post *v*. Pre) for total, LDL- and HDL-cholesterol or TAG were observed within each group or between the two groups (Table 4).

**Table 4:** Lipid profile, inflammatory and thrombogenic markers, and oxidative status before and after 6-week consumption of strawberry and cranberry polyphenols (SCP) or Control in insulin-resistant human subjects (Mean values with their standard errors)

	SCP (*n* 20)					Control (*n* 21)					
	Pre		Post			Pre		Post			
Variables	Mean	sem	Mean	sem	*P*_1_ *	Mean	sem	Mean	sem	*P*_2_ *	*P* †
Cholesterol (mmol/l)											
Total	5·70	0·17	5·60	0·19	0·52	5·37	0·22	5·45	0·20	0·45	0·33
HDL	1·25	0·05	1·26	0·06	0·68	1·33	0·05	1·37	0·06	0·10	0·40
LDL	3·52	0·17	3·51	0·17	0·97	3·20	0·15	3·37	0·17	0·05	0·32
Total cholesterol:HDL-cholesterol ratio	4·76	0·27	4·62	0·24	0·16	4·12	0·20	4·08	0·17	0·60	0·41
TAG (mmol/l)	2·03	0·24	1·82	0·21	0·06	1·73	0·26	1·56	0·18	0·07	0·99
hsCRP (mg/l)‡	3·6	0·7	3·0	0·6	0·13	5·4	2·9	3·0	0·6	0·69	0·53
TNF-*α* (ng/l)§	4·4	0·4	4·0	0·4	0·06	4·3	0·2	4·0	0·3	0·32	0·69
IL-6 (ng/l)§	4·9	0·4	4·8	0·6	0·99	5·6	1·0	4·9	0·8	0·24	0·23
HMW adiponectin (µg/ml)§	6·83	1·10	5·91	0·93	0·15	7·15	1·15	6·73	1·20	0·61	0·65
PAI-1×10^3^ (ng/l)§	30·5	2·7	28·5	3·1	0·46	30·0	2·5	27·3	3·5	0·41	0·87
RANTES (µg/l)‡	3·21	0·39	3·15	0·44	0·86	3·06	0·40	2·77	0·37	0·53	0·71
Oxidised-LDL (U/l)‡	96·5	6·2	92·9	5·6	0·14	79·7	5·4	80·2	4·8	0·83	0·22
FRAP (µm Fe^2+^/l)‡	1191	58	1237	65	0·37	1135	36	1189	41	0·07	0·70

hsCRP, high-sensitivity C-reactive protein; HMW, high molecular weight; PAI-1, plasminogen activator inhibitor-1; RANTES, regulated on activation, normal T cell expressed and secreted; FRAP, ferric reducing antioxidant power.

* *P* value obtained from paired *t* test to compare changes from baseline (Post *v*. Pre) within the SCP (*P*_1_) and Control (*P*_2_) groups.

† *P* value represents between-treatment comparison of changes from baseline (Post *v*. Pre), assessed by PROC MIXED ANOVA. Due to significant correlations between lipid and oral glucose tolerance test variables, a Bonferroni correction was applied for these variables, defining level of statistical significance at *P*<0·004.

‡ *n* 38–39 (SCP *n* 18–20; Control *n* 18–21).

§ *n* 33 (SCP *n* 15; Control *n* 18).

### Inflammatory, thrombogenic and oxidative markers

The effects of SCP on inflammatory and oxidative stress markers are shown in Table 4. No differences in changes from baseline (Post *v*. Pre) for pro-inflammatory cytokines, hsCRP, HMW adiponectin, PAI-1, oxidised-LDL, RANTES or total antioxidant capacity of plasma (FRAP) were observed within each group or between the two groups.

### Phenolic composition and bioavailability of experimental beverages

**Fig. 5:**
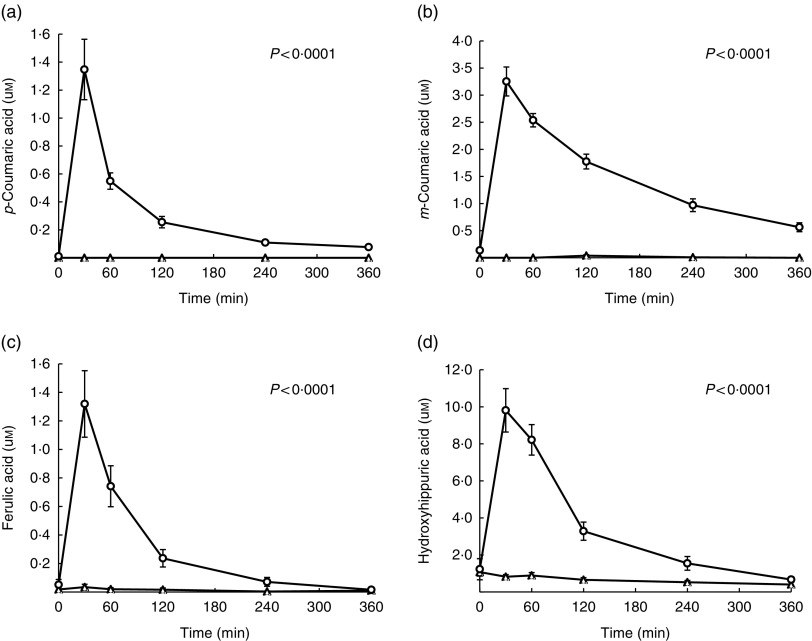
Evolution of post-ingestion plasma concentrations of phenolic metabolites. (a) *p*-coumaric acid, (b) *m*-coumaric acid, (c) ferulic acid, (d) hydroxyhippuric acid. Values are mean of replicates (*n* 17), with their standard errors. Welch’s *t* test (correcting for unequal variance) when data were assumed to be normally distributed and the non-parametric Mann–Whitney test otherwise, showed significantly higher concentrations of phenolic metabolites following the consumption of strawberry and cranberry polyphenols (SCP) relative to Control. A Bonferroni correction (*P*<0·0025) was performed. *P*<0·0001 for each phenolic metabolite. 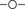, SCP; 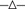, Control.

As shown in Table 1, SCP-containing and SCP-free Control beverages differed significantly in terms of phenolic content. SCP contained four times more proanthocyanidins and six times more phenolic acids. In particular, SCP were characterised by a very high content of coumaric acids: *p*-coumaric acid, *m*-coumaric acid and coumaroyl glucoside. At the 30-d midpoint of the daily consumption of the beverages, about twenty phenolic metabolites were identified in the plasma of volunteers. They are present in circulation as conjugate metabolites and microbial degradation products, because the native phenolic compounds are normally extensively metabolised. Among these, *p*-coumaric acid, *m*-coumaric acid, ferulic acid and hydroxyhippuric acid, whose absorption kinetics is presented in Fig. 5, were detected in significantly higher concentration following the consumption of SCP, relative to Control (respective AUC of 17·4 (sem 1·8) *v*. 0, 85·9 (sem 5·3) *v*. 0·7 (sem 0·3), 20·3 (sem 3·9) *v*. 0·9 (sem 0·4), 204·6 (sem 25·4) *v*. 38·6 (sem 6·9); *P*<0·0001). A significant negative correlation was found between plasma concentration of *p*-coumaric acid (AUC 0–30 min) and the percentage change in IAUC (before and after treatment) of the above-mentioned C-peptide response (first 30 min of the OGTT) (*P*=0·0046, *r* ^2^ 0·34). No correlations were reported between the remaining metabolites and the parameters related to insulin sensitivity and the first 30 min OGTT.

## Discussion

This study investigated the impact of daily consumption of a 333 mg SCP blend on insulin sensitivity in insulin-resistant, non-diabetic subjects over a period of 6 weeks. The main outcomes of this study are: (1) an improvement in insulin sensitivity, as assessed by hyperinsulinaemic-euglycaemic clamp and (2) the prevention of further compensatory insulin secretion, as shown by a lack of increase in the early C-peptide response during an OGTT. Analysis of plasma phenolic metabolites also revealed that the most abundant phenolic acids identified with SCP intake were *p*-coumaric acid, *m*-coumaric acid, ferulic acid and hydroxyhippuric acid. Interestingly, plasma *p*-coumaric acid was inversely related to the early C-peptide response during OGTT.

The hyperinsulinaemic-euglycaemic clamp is the gold standard and reference technique for assessing insulin sensitivity when whole-body insulin sensitivity is the primary outcome^(^ ^20^ ^)^. It is a steady-state technique that requires a constant insulin infusion. It is the most reliable technique in a clinical study like ours with relatively low number of subjects. Although indirect OGTT-derived indices (e.g. Matsuda index and ISI) may be of particular interest in prospective studies with large cohorts, they have potential limitations in reproducibility due to intra-individual variation in plasma glucose and insulin responses during the OGTT^(^ ^34^ ^)^. With regard to the indirect indices based on fasting levels of glucose and insulin (e.g. HOMA-IR and QUICKI), they tend to assess hepatic insulin resistance rather than peripheral and whole-body insulin sensitivity^(^ ^35^ ^)^.

Our study demonstrates an improvement in insulin sensitivity following the consumption of SCP compared with Control. These results are in good agreement with those of Stull *et al*.^(^ ^22^ ^)^ who observed a 22 % increase in insulin sensitivity, assessed with the hyperinsulinaemic-euglycaemic clamp technique, following a 6-week daily dietary supplementation with whole blueberries in obese, non-diabetic, and insulin-resistant human subjects, and those of Hokayem *et al*.^(^ ^23^ ^)^ who noted that the negative effects of fructose used to develop insulin resistance were counteracted by grape polyphenol supplementation in a double-blind controlled trial. It is noteworthy that our dose of polyphenols (a total of 333 mg of combined SCP/d), which is a much lower dose than those used by Stull *et al*.^(^ ^22^ ^)^ (1462 mg polyphenols from blueberry powder/d) and Hokayem *et al*.^(^ ^23^ ^)^ (2 g from grape polyphenols/d), can exert a comparable effect on insulin sensitivity. The present results with SCP are also in good agreement with those reported by Edirisinghe *et al.* ^(^ ^36^ ^)^ who observed a reduction in postprandial insulin response in overweight adults after a single consumption of a high-carbohydrate, moderate-fat meal with a strawberry beverage containing 10 g of strawberries in a freeze-dried form (95 mg of polyphenols). Furthermore, Park *et al*.^(^ ^37^ ^)^ observed a reduction in systolic and diastolic blood pressure concomitant with a trend to improve fasting insulin and insulin sensitivity in subjects with pre-hypertension consuming a grape seed extract-containing beverage (528 mg of polyphenols). Most of these subjects were hyperinsulinaemic or insulin resistant. This is of interest in the present study, given the evidence supporting a link between hypertension and insulin resistance through insulin mediated signalling pathways^(^ ^38^ ^)^ and NO production^(^ ^39^ ^)^. Similarly, Rodriguez-Mateos *et al*.^(^ ^40^ ^)^ reported that endothelial function (at 1 h) in healthy men increased in a dose-dependent manner up to an intake of 766 mg polyphenols from a blueberry drink and reached a plateau at higher doses. Therefore, the present results together with those from the three latter studies suggest that polyphenol doses lower than 800 mg may offer metabolic benefits. Of note, the beneficial effect of SCP on insulin sensitivity observed in the present study cannot be explained by variations in energy and macronutrient intake, body weight or body fat mass since no changes in these parameters were seen between the two groups.

The progression from normal glucose tolerance to type 2 diabetes is characterised by both an increase in insulin resistance and a decrease in insulin secretion caused by *β*-cell dysfunction. Insulin resistance is defined as decreased tissue sensitivity to insulin to stimulate glucose uptake and utilisation. In the early stages of insulin resistance, plasma glucose is maintained at normal levels by a compensatory increase in insulin secretion, the first abnormality being an increase in first-phase insulin secretion by pancreatic *β*-cells^(^ ^41^ ^)^. But when *β*-cell compensation fails, fasting plasma glucose levels rise (IFG), leading to IGT and eventually type 2 diabetes^(^ ^42^ ^)^. In the context of the present study, the beverage rich in polyphenols prevented a further elevation in early-phase insulin release, as indicated by C-peptide levels, and prevented the overall increase of insulin secretion, suggesting that the improvement in insulin sensitivity after consumption of the SCP beverage may have precluded a further compensatory increase in insulin secretion. A reducing effect of a polyphenol-rich cranberry extract has already been observed on C-peptide levels in high-fat/high-sucrose-fed mice^(^ ^43^ ^)^. However, in humans, to the best of our knowledge, this is the first report showing a beneficial effect of a SCP extract on C-peptide response during an OGTT.

In this study, we found differences between the SCP and the Control groups in the plasma levels of four polyphenolic compounds and metabolites (*p*-coumaric acid, *m*-coumaric acid, ferulic acid, hydroxyhippuric acid). These levels were found in the same low micromolar range as that reported by Feliciano *et al.* ^(^ ^44^ ^)^ and Park *et al.* ^(^ ^37^ ^)^, where physiological and metabolic bioactivity is probable^(^ ^45^ ^)^. Particularly of interest, an increased plasma concentration of *p*-coumaric acid during the 30 min post-consumption was found to significantly correlate with a reduced secretion of C-peptide compared with the Control during the early phase of the OGTT response (*P*=0·0046, *r* ^2^ 0·34). Although a direct effect of *p*-coumaric acid on insulin sensitivity cannot be confirmed by these results, this compound was recently found to stimulate AMP-activated protein kinase phosphorylation, leading to increased glucose uptake in L6 myocytes^(^ ^46^ ^)^. *p*-Coumaric acid was also found to improve glucose uptake *in vitro* through synergistic interactions with a commercial oral hypoglycaemic drug (thiazolidinedione)^(^ ^47^ ^)^. Moreover, since only 5–10 % of ingested phenolic compounds are assumed to be absorbed^(^ ^48^ ^)^, a large amount proceeds to the large intestine (especially polymeric forms such as proanthocyanidins) where they can exert an activity. Indeed, we recently showed that cranberry polyphenols can improve insulin sensitivity in high-fat-fed mice, leading to reduced inflammation in both intestinal and hepatic tissues, through modulation of gut microbiota^(^ ^43^ ^)^. In this latter study, changes in gut microbiota were detected following 5 weeks of supplementation, suggesting that 6 weeks of supplementation in humans was very likely a period long enough to modulate their intestinal microbiota. SCP may also improve insulin sensitivity by increasing insulin signalling and glucose transport in skeletal muscle cells. Indeed, Nizamutdinova *et al*.^(^ ^17^ ^)^ showed that anthocyanins administrated by gavage can improve insulin signalling by stimulating tyrosine phosphorylation of the insulin receptors and by increasing the expression of GLUT4 in muscle of streptozotocin-diabetic rats.

Whereas our study provides evidence for the beneficial effect of SCP treatment on insulin sensitivity, this finding was not associated with a decrease in other markers of cardiovascular risk. Nutritional studies are somewhat contradictory regarding the effects of strawberries and cranberries on cardiometabolic markers such as plasma lipids, oxidative stress, antioxidant capacity and inflammation. Indeed, lipid changes seen in our study contrast with those reported by Basu *et al.* ^(^ ^7^ ^)^ and Lee *et al.* ^(^ ^49^ ^)^ who observed a decrease in total cholesterol and LDL-cholesterol in human subjects consuming either freeze-dried strawberry powder^(^ ^8^ ^)^ or cranberry extract^(^ ^49^ ^)^. Similarly, our findings on inflammatory and oxidative markers do not agree with those of Moazen *et al.* ^(^ ^50^ ^)^ who observed a decrease in plasma CRP and oxidised-LDL after administration of freeze-dried strawberries, and those of Ruel *et al.* ^(^ ^9^ ^,^ ^11^ ^,^ ^51^ ^)^ who observed an increase of plasma HDL-cholesterol, antioxidant capacity and a decrease in oxidised-LDL after consumption of low-energy cranberry drink. Additionally, some clinical studies investigating the effects of approximately 300 mg polyphenols from freeze-dried strawberry powder reported reducing effects on fasting oxidised LDL for 6 weeks^(^ ^9^ ^)^, and postprandial CRP and IL-6^(^ ^36^ ^)^ in an overweight, hyperlipidaemic population. However, other clinical studies using freeze-dried strawberry powder or reduced-energy cranberry juice have demonstrated, as in our study, a lack of effect on antioxidant status^(^ ^52^ ^)^, on CRP, IL-6 and TNF-*α* ^(^ ^52^ ^,^ ^53^ ^)^.

Discrepancies between our results on lipids and inflammatory and oxidative markers and those of the above-cited studies^(^ ^8^ ^10^ ^,^ ^12^ ^,^ ^36^ ^,^ ^49^ ^–^ ^51^ ^)^ may stem from the delivery form of strawberry (freeze-dried form *v*. SCP extract) or cranberry (juice, dried fruit forms or SCP extract), the experimental design (acute *v*. longer term), the polyphenol dose or the population studied. In the present study, the SCP were a SCP enriched extract providing 333 mg/d of polyphenols and was devoid of fibres, sugars, minerals and vitamins. On the one hand, this amount of polyphenols, which corresponds to the quantity supplied by about 120 g of fresh fruits, is equivalent to approximately one-fourth the level of polyphenols present in the freeze-dried strawberry powder used by Basu *et al.* ^(^ ^7^ ^8^ ^)^ and Moazen *et al.* ^(^ ^50^ ^)^ (1001–2006 mg polyphenols/d) and in the low-energy cranberry juice used in previous studies^(^ ^9^ ^–^ ^12^ ^,^ ^15^ ^,^ ^51^ ^)^. On the other hand, the key difference between the SCP extract used in the present study and these other sources of polyphenols is the lack of fermentable fibres. It is well recognised that strawberries^(^ ^54^ ^)^ and cranberries^(^ ^55^ ^)^ contain significant amounts of fermentable soluble and insoluble fibres that may reduce lipids, inflammatory and oxidative markers^(^ ^56^ ^)^. Therefore, the lack of dietary fibre in our extract is among the plausible explanations for the absence of an SCP effect on lipids, inflammatory and oxidative markers in the current study. Moreover, the present results and those from a study by Hokayem *et al.* ^(^ ^23^ ^)^ who used a berry (grape) polyphenol extract, suggest that the various phenol compounds present in berries may specifically improve insulin sensitivity in humans.

The participants in this study were insulin resistant and included both sexes and a relatively broad age range (40–70 years). Given the free-living nature of the study, the results can most likely apply to an overweight adult pre-diabetic population. Despite the parallel arm design and polyphenol content of the beverage used, the present study did allow sufficient power to detect statistical differences on the primary endpoint of insulin sensitivity. However, this study was not sufficiently powered to detect differences on secondary endpoints, in particular hsCRP, TNF-*α* and oxidised-LDL. Yet, it would have been interesting to obtain muscle and adipose tissue biopsies to test whether SCP reduced inflammation in these tissues. Such biopsies may have also allowed us to ascertain the identity of the molecules involved in cellular insulin signalling. Nonetheless, considering the robust nature of our randomised, controlled, double-blind study, it is likely that the consumption of SCP resulted in the improvement in the hyperinsulinaemic-euglycaemic clamp insulin sensitivity and OGTT parameters. Finally, since this is a proof-of-concept study, it will be necessary to consider longer-term interventions in larger populations of subjects to confirm the results and expand upon the potential role of SCP in preventing or delaying the onset of type 2 diabetes.

In conclusion, our data indicate that 6-week consumption of 333 mg polyphenols from strawberries and cranberries may improve insulin sensitivity and prevent an increase in compensatory insulin secretion without affecting plasma lipids, CRP, pro-inflammatory cytokines and antioxidant capacity. Controlled dose–response trials are needed to ascertain the lower and upper range of activity of these polyphenols, as well as larger and longer-term studies.

## Supplementary material

For supplementary material accompanying this paper visit http://dx.doi.org/10.1017/S0007114517000393. click here to view supplementary material
