# Supplementary material for: Strawberry and cranberry polyphenols improve insulin sensitivity in insulin-resistant, non-diabetic adults: a parallel, double-blind, controlled and randomised clinical trial
Source: Br J Nutr. 2017 Mar 14;117(4):519–31. doi: 10.1017/S0007114517000393 (PMC5426341; doi:10.1017/S0007114517000393)
Supplement: Supplementary file 1 [file S0007114517000393sup001.zip › S0007114517000393sup001.docx]

**Online supporting material**

Assessed for eligibility (*n* 116)

Randomized (*n* 50)

Excluded (*n* 66)

- Not meeting inclusion criteria (*n* 59)
- Declined to participate (*n* 7)

## Enrolment

## Allocation

**SCP**

Allocated to intervention (*n* 24)

- Received allocated intervention (*n* 24)
- Did not receive allocated intervention (*n* 0)

**Control**

Allocated to intervention (*n* 26)

- Received allocated intervention (*n* 24)
- Did not receive allocated intervention (*n* 2)

Reason:

Normalization of insulin sensitivity before starting the intervention

## Follow-up

Lost to follow-up (personal reasons) (*n* 1)

Lost to follow-up (personal reasons) (*n* 1)

1

## Analysis

Completed the intervention (*n* 23)

- Excluded from analysis (*n* 3)

Reasons:

No longer met the inclusion criteria:

Weight change ≥ 2 kg (*n* 1)

Medical reasons (*n* 1)

Sub-optimal compliance (*n* 1)

Completed the intervention (*n* 23)

- Excluded from analysis (*n* 2)

Reasons:

No longer met the inclusion criteria:

Weight change ≥ 2 kg (*n* 1)

Medical reasons (*n* 1)

**Analysed (*n* 21)**

**Analysed (*n* 20)**

**Supplemental Fig. 1.** Participant Flow Diagram.

SCP, strawberry and cranberry polyphenols.
