# Supplementary material for: Strawberry and cranberry polyphenols improve insulin sensitivity in insulin-resistant, non-diabetic adults: a parallel, double-blind, controlled and randomised clinical trial
Source: Br J Nutr. 2017 Mar 14;117(4):519–31. doi: 10.1017/S0007114517000393 (PMC5426341; doi:10.1017/S0007114517000393)
Supplement: Supplementary file 1 [file S0007114517000393sup001.zip › S0007114517000393sup002.pptx]

## Slide 1
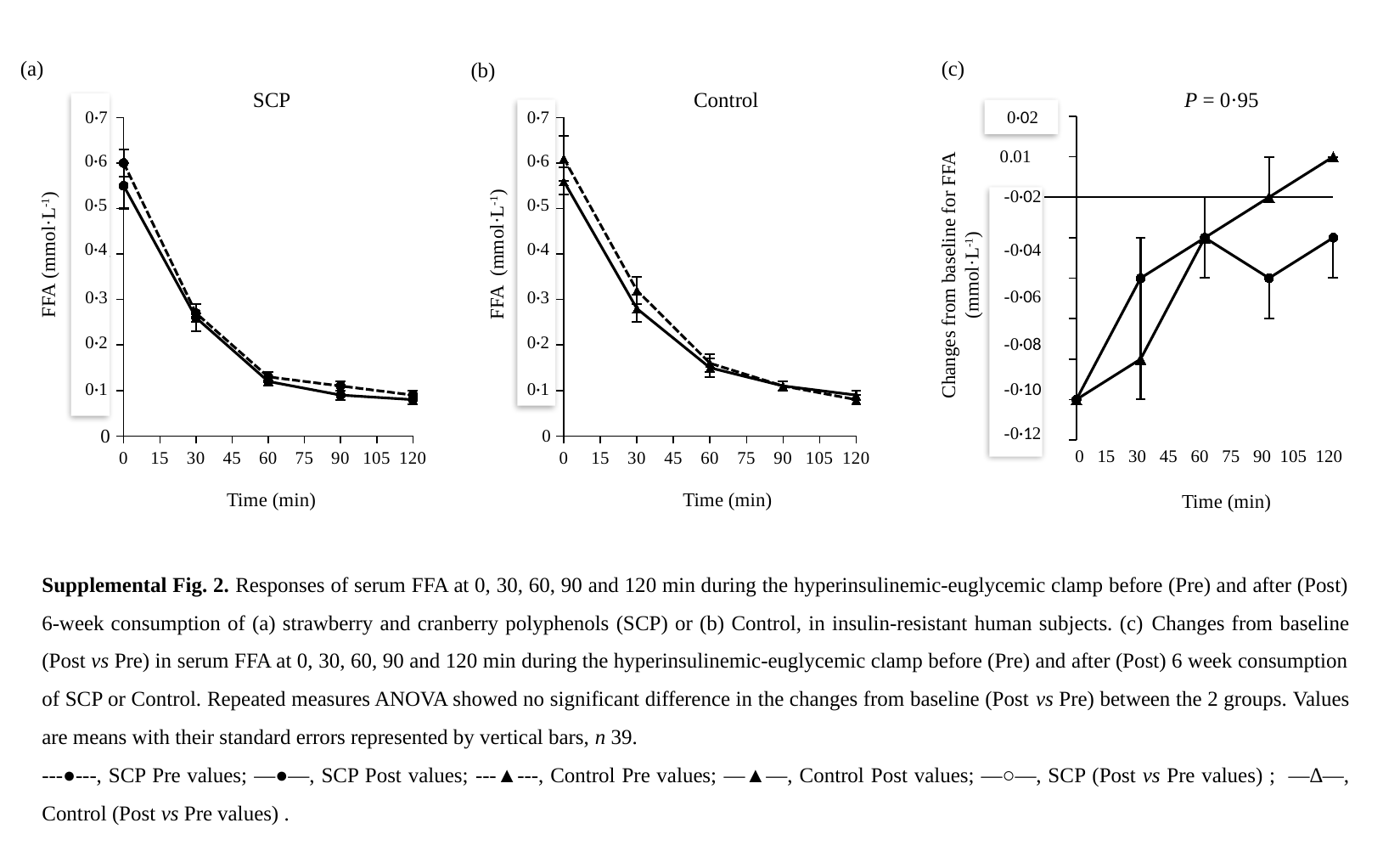

(a)
(c)
(b)
SCP
Control
### Chart
| Category | | |
|---|---|---|
### Chart
| Category | | |
|---|---|---|FFA (mmol·L-1)
FFA (mmol·L-1)
Changes from baseline for FFA (mmol·L-1)
Time (min)
Time (min)
Time (min)
### Chart
| Category | | |
|---|---|---| 0 15 30 45 60 75 90 105 120
Supplemental Fig. 2. Responses of serum FFA at 0, 30, 60, 90 and 120 min during the hyperinsulinemic-euglycemic clamp before (Pre) and after (Post) 6-week consumption of (a) strawberry and cranberry polyphenols (SCP) or (b) Control, in insulin-resistant human subjects. (c) Changes from baseline (Post vs Pre) in serum FFA at 0, 30, 60, 90 and 120 min during the hyperinsulinemic-euglycemic clamp before (Pre) and after (Post) 6 week consumption of SCP or Control. Repeated measures ANOVA showed no significant difference in the changes from baseline (Post vs Pre) between the 2 groups. Values are means with their standard errors represented by vertical bars, n 39.
---●---, SCP Pre values; —●—, SCP Post values; ---▲---, Control Pre values; —▲—, Control Post values; —○—, SCP (Post vs Pre values) ; —∆—, Control (Post vs Pre values) .
P = 0·95
 0·7
 0·02
 0·7
 0·6
 0·6
 -0·02
 0·5
 0·5
 0·4
 0·4
 -0·04
 -0·06
 0·3
 0·3
 0·2
 0·2
 -0·08
 0·1
 0·1
 -0·10
 -0·12
