# Supplementary material for: Strawberry and cranberry polyphenols improve insulin sensitivity in insulin-resistant, non-diabetic adults: a parallel, double-blind, controlled and randomised clinical trial
Source: Br J Nutr. 2017 Mar 14;117(4):519–31. doi: 10.1017/S0007114517000393 (PMC5426341; doi:10.1017/S0007114517000393)
Supplement: Supplementary file 1 [file S0007114517000393sup001.zip › S0007114517000393sup003.docx]

**Online supporting material**

**Supplemental Table 1.** Dietary energy and macronutrient intake assessed by FFQ before and during 6-week consumption of SCP or Control in insulin-resistant human subjects ^*^

(Mean values with their standard errors)

SFA, saturated fatty acids; MUFA, Monounsaturated fatty acids; PUFA, Polyunsaturated fatty acids; SCP, strawberry and cranberry polyphenols.

|  | SCP (*n* 20) | | | | |  | Control (*n* 21) | | | | |  |
| --- | --- | --- | --- | --- | --- | --- | --- | --- | --- | --- | --- | --- |
|  | Pre | |  | Post | |  | Pre | |  | Post | |  |
| Variables | Mean | SEM |  | Mean | SEM |  | Mean | SEM |  | Mean | SEM | *P* Value |
| Energy (kcal) | 2271 | 196 |  | 2284 | 179 |  | 2519 | 243 |  | 2206 | 174 | 0·27 |
| Carbohydrate (g) | 257 | 23 |  | 270 | 26 |  | 270 | 19 |  | 247 | 18 | 0·23 |
| Protein (g) | 96 | 9 |  | 98 | 7 |  | 118 | 16 |  | 101 | 10 | 0·12 |
| Lipid (g) | 96 | 8 |  | 95 | 7 |  | 107 | 14 |  | 93 | 8 | 0·32 |
| Alcohol (g) | 9 | 3 |  | 4 | 1 |  | 9 | 3 |  | 6 | 3 | 0·91 |
| SFA (g) | 30 | 3 |  | 30 | 3 |  | 37 | 5 |  | 32 | 4 | 0·27 |
| MUFA (g) | 39 | 3 |  | 38 | 3 |  | 43 | 6 |  | 37 | 3 | 0·35 |
| PUFA (g) | 19 | 2 |  | 19 | 2 |  | 19 | 2 |  | 17 | 1 | 0·43 |

^*^ PROC GLM ANOVA test showed no significant differences in the changes from baseline (Post *vs* Pre) between the 2 groups.

**Online supporting material**

**Supplemental Table 2.** Anthropometric measures and blood pressure before and after 6-week consumption of SCP or Control in insulin-resistant human subjects^*^

(Mean values with their standard error)

|  | SCP (*n* 20) | | | | |  | Control (*n* 21) | | | | |  |
| --- | --- | --- | --- | --- | --- | --- | --- | --- | --- | --- | --- | --- |
|  | Pre | |  | Post | |  | Pre | |  | Post | |  |
| Variables | Mean | SEM |  | Mean | SEM |  | Mean | SEM |  | Mean | SEM | P value |
| Systolic blood pressure (mmHG) | 117 | 3 |  | 116 | 3 |  | 123 | 3 |  | 120 | 3 | 0·72 |
| Diastolic blood pressure (mmHG) | 70 | 2 |  | 68 | 2 |  | 73 | 2 |  | 71 | 2 | 0·92 |
| Weight (kg) | 85 | 3 |  | 85 | 3 |  | 85 | 3 |  | 85 | 3 | 0·62 |
| BMI (kg/m2) | 31 | 1 |  | 31 | 1 |  | 31 | 1 |  | 31 | 1 | 0·63 |
| Waist circumference (cm) | 104 | 3 |  | 103 | 3 |  | 104 | 2 |  | 103 | 2 | 0·77 |
| Hip circumference (cm) | 111 | 2 |  | 110 | 2 |  | 111 | 2 |  | 110 | 2 | 0·59 |
| Waist to hip ratio | 0·9 | 0·0 |  | 0·9 | 0·0 |  | 0·9 | 0·0 |  | 0·9 | 0·0 | 0·61 |

SCP, strawberry and cranberry polyphenols.

^*^ PROC MIXED ANOVA test showed no significant differences in the changes from baseline (Post *vs* Pre ) between the 2 groups.
